# Spatiotemporal trends and driving factors of gonorrhea incidence in China: a 20-year analysis (2003–2022)

**DOI:** 10.3389/fpubh.2025.1699572

**Published:** 2026-01-12

**Authors:** Ke Hu, Chaojie Li, Xingjin Yang, Shuiping Ou, Xing Zhang, Di Xiao, Mingyang Yu

**Affiliations:** 1 Xiamen Haicang Hospital, Xiamen, Fujian, China; 2 Xingtai Center for Disease Control and Prevention, Xingtai, Hebei, China; 3 QianDongNanZhou Center for Disease Control and Prevention, QianDongNanZhou, Guizhou, China; 4 Honwing Pharma (Guizhou) Company Limited, QianDongNanZhou, Guizhou, China; 5 Nanjing Lishui Dongping Street Health Center, Nanjing, Jiangsu, China; 6 Community Health Service Center of Jiuxian Tongliang District, Chongqing, China; 7 Fuwai Central China Cardiovascular Hospital, Zhengzhou, Henan, China

**Keywords:** driving factors, geographically and temporally weighted regression, gonorrhea incidence, spatiotemporal dynamics, spatiotemporal scan

## Abstract

**Introduction:**

Gonorrhea represents a major global public health challenge, demonstrating significant spatiotemporal heterogeneity across China. Therefore, spatiotemporal analytical approaches are required to better understand gonorrhea incidence patterns.

**Methods:**

Employing an innovative methodological framework that combines spatiotemporal scan statistics with Geographically and Temporally Weighted Regression (GTWR), this study systematically examines gonorrhea incidence dynamics across mainland China from 2003 to 2022, with particular focus on exploring the impacts of healthcare resources, economic development, education levels, and demographic structure on gonorrhea incidence.

**Results:**

Our comprehensive analysis reveals three key findings: First, while national incidence rates declined significantly from 20.64 cases per 100,000 population in 2004 to 5.99 in 2022, the rate of decrease slowed markedly after 2013, suggesting a stabilization phase in prevention effectiveness. Second, spatial analysis identified distinct epidemiological patterns, with persistent high-risk clusters in the southeastern region contrasting with emerging hotspots in western provinces, reflecting China’s uneven regional development. Third, our GTWR modeling uncovered complex driver dynamics: healthcare resources showed temporally weakening effects, economic development shifted from protective to risk-enhancing effects, education maintained stable protective effects, and demographic influences exhibited significant spatial variation – particularly the increasing association between male predominance and higher incidence in southern provinces.

**Conclusion:**

These findings enhance gonorrhea surveillance precision and inform targeted regional prevention approaches, while providing a scientific basis for optimizing public health resource allocation.

## 1 Introduction

Gonorrhea, as a significant global public health issue, exhibits notable spatiotemporal heterogeneity in China. Multiple studies have demonstrated distinct spatial clustering and temporal fluctuations in gonorrhea incidence across Chinese provinces, with high-risk clusters concentrated in southern and eastern regions like Zhejiang and Guangdong (2004–2014) showing seasonal peaks in the third quarter (1, 2). Persistent urban hotspots were observed Hangzhou and Jiaxing in Zhejiang (2016–2020), with higher incidence in northern and central areas than southern regions (3). Although national rates declined post-2004, periodic surges occurred (2015–2018) with consistent seasonal peaks in July and December (4, 5), indicating transmission may be influenced by socioeconomic, demographic, climate, and other factors, warranting further spatiotemporal analysis.

Gonorrhea incidence shows significant spatial variation linked to sociodemographic and healthcare factors. Studies reveal higher risks in areas with greater GDP per capita, lower physician density, skewed gender ratios (male-dominated), and elevated divorce rates (2). Urbanization and population density may increase transmission through expanded sexual networks, while education and household size could reduce risk via behavioral changes (6, 7). Notably, the effects of these factors may exhibit spatial non-stationarity. For instance, higher GDP per capita showed stronger associations with gonorrhea risk in southern and eastern China’s high-risk clusters compared to other regions (2), necessitating analytical methods that account for spatial heterogeneity.

Traditional linear regression models are limited in analyzing gonorrhea influencing factors due to their inability to account for spatial non-stationarity (8). While spatial regression models incorporate spatial dependencies, they still assume spatially invariant relationships (9). Geographically weighted regression addresses spatial heterogeneity through location-varying coefficients but ignores temporal dynamics (10). Similarly, the Time Weighted Regression (TWR) model effectively addresses temporal non-stationarity and local variations over time, but does not account for spatial heterogeneity. Spatial panel models include time-fixed effects but maintain a global framework, limiting their ability to detect localized spatiotemporal parameter variations (11).

In contrast, geographically and temporally weighted regression (GTWR) incorporates both spatial and temporal weighting kernels to capture spatial heterogeneity and its temporal dynamics—such as the waning effect of public health measures over time (12). The spatiotemporal weight matrix in GTWR also helps distinguish long-term trends from localized anomalies, providing a fuller understanding of disease determinants (13–16). Empirical studies confirm that GTWR outperforms traditional spatial and spatial panel models in both fit and explanatory power, particularly for infectious diseases with strong spatiotemporal variation like gonorrhea (17).

By integrating spatiotemporal scan statistics with GTWR modeling, this study aims to elucidate the spatiotemporal variation patterns of gonorrhea incidence in China and identify key influencing factors. The identification of high-risk clusters and spatially non-stationary key determinants will provide scientific evidence for formulating regionally tailored prevention strategies.

## 2 Methods

### 2.1 Data sources and variable selection

The selection of independent variables for this study is theoretically grounded in the recognized associations between sociodemographic, economic, and healthcare factors and gonorrhea transmission dynamics, as discussed in the introduction. Guided by this established framework and data availability, eight provincial-level indicators were chosen to capture these multidimensional influences (see Table 1). Specifically, healthcare resource and capacity are represented by the ‘Number of licensed physicians per 1,000 population’ and the ‘Hospital bed occupancy rate’. Economic development is measured by ‘GDP per capita’. The ‘Average years of schooling’ serves as a proxy for education level. Population structure and mobility dynamics are captured by ‘Population density’, ‘Average household size’, ‘Sex ratio’, and the ‘Urbanization rate’. This selection allows for an investigation of the potential spatial non-stationarity in the effects of these determinants on gonorrhea incidence.

**Table 1:** Key factors selected for analysis.

Categories	Factors
Healthcare resources	Number of licensed physicians per 1,000 population
	Hospital bed occupancy rate
Economic development	GDP per capita
Education level	Average years of schooling
Population structure	Population density
	Average household size
	Sex ratio
	Urbanization Rate

This study analyzed gonorrhea incidence patterns across mainland China’s 31 provincial-level administrative divisions, excluding Hong Kong, Macau, and Taiwan. All data were obtained from authoritative statistical sources: gonorrhea incidence rates from 2003 to 2022 were extracted from the China Health Statistical Yearbook, while explanatory variables for the corresponding years were sourced from the officially published China Statistical Yearbook.

### 2.2 Spatial distribution analysis

This study employed spatial visualization techniques to systematically analyze gonorrhea incidence patterns across China’s 31 provincial-level regions from 2003 to 2022 using five-year intervals (2003, 2008, 2013, 2018, and 2022). The five-year interval approach was strategically designed to balance data reliability with meaningful trend analysis by reducing short-term fluctuations while maintaining alignment with China’s public health planning cycles and preserving sufficient temporal resolution to observe epidemiological transitions and spatial pattern evolution.

### 2.3 Global spatial autocorrelation test

Moran’s I index was utilized to quantitatively evaluate the spatial autocorrelation of gonorrhea incidence. The calculation formula for Moran’s *I* is as follows Equation 1(18):


$$I=\frac{n\sum_{i=1}^{n}\sum_{j=1}^{n}W_{\mathrm{ij}}(x_{i}-\bar{x})(x_{j}-\bar{x})}{\sum_{i=1}^{n}\sum_{j=1}^{n}W_{\mathrm{ij}}\sum_{i=1}^{n}{\left(x_{i}-\bar{x}\right)}^{2}}$$ (1)

The index ranges from −1 to 1, where *n* represents the number of samples, *W*_ij_ denotes the elements of the spatial weight matrix, *x*_i_ and *x*_j_ are the observed values, and $\bar{x}$ is the mean value. The interpretation of Moran’s *I* is as follows: *I* > 0 indicates positive spatial autocorrelation (clustering of similar values), *I* < 0 indicates negative spatial autocorrelation (adjacent high and low values), and *I* ≈ 0 suggests random distribution. All results were verified for significance using Z-tests (*p* < 0.05) (19).

### 2.4 Spatiotemporal scan analysis

The study used spatiotemporal scan statistics to analyze gonorrhea incidence patterns across China’s 31 provinces (2003–2022). This method was chosen for its capacity to precisely identify the location, spatial extent, and temporal duration of incidence clusters while providing statistical inference. Under a Poisson model, the analysis employed dynamically adjustable cylindrical scanning windows with the maximum spatial cluster size set to include up to 50% of the total population at risk and the maximum temporal window limited to 50% of the study period (i.e., 10 years) (20). The log likelihood ratio (LLR) assessed cluster significance (*p* < 0.05) (21), while relative risk (RR) quantified risk levels (22). Clusters were classified as primary (highest LLR, *p* < 0.01, indicating strong aggregation) or secondary (elevated LLR, *p* < 0.05, reflecting moderate risk) (23). The population at risk, defined as the annual average population of each province, was incorporated into the model to calculate expected case counts, ensuring the identified clusters reflected elevated incidence risk rather than mere population density variations. Results were validated by 999 Monte Carlo simulations, confirming robust detection of spatiotemporal patterns (24).

### 2.5 Regression models

#### 2.5.1 Multiple linear regression (MLR)

This study examined gonorrhea incidence (*Y*) using MLR after evaluating multicollinearity through Variance Inflation Factor (VIF) analysis (threshold = 5) (25). The model was formulated as Equation 2:


$$Y=\beta_{0}+\sum \beta_{i}X_{i}+\varepsilon$$ (2)

where *ε* follows *N*(0,σ^2^), *X*ᵢ are selected predictors (VIF < 5), *β*₀ is intercept, *β*ᵢ are coefficients. This global approach estimates constant spatial–temporal effects.

#### 2.5.2 Time-weighted regression (TWR) model

TWR model was developed with time-varying coefficients Equation 3:


$$Y(t)=\beta_{0}(t)+\sum \beta_{i}(t)X_{i}+\varepsilon (t)$$ (3)

The temporal weights were defined by a Gaussian kernel function Equation 4:


$$w(t_{i},t_{j})=\exp[-{\left(t_{i}-t_{j}\right)}^{2}/h_{t}^{2}]$$ (4)

where *h_t_* represents the temporal bandwidth parameter optimized through AIC_c_ minimization. This formulation enables smooth temporal variations in coefficients *β*ᵢ(t), effectively capturing the dynamic relationships between gonorrhea incidence (*Y*) and predictors (*X*ᵢ) while preserving model generalizability (26).

#### 2.5.3 Geographically weighted regression (GWR) model

The GWR model applied with a fixed Gaussian kernel to analyze spatial non-stationarity (18). The spatial weight function is defined as Equation 5:


$$w(d_{\mathrm{ij}})={\left[-{\left(1-(d_{\mathrm{ij}}/h_{s})\right)}^{2}\right]}^{2}$$ (5)

when the distance *d*_ij_ is within bandwidth *h*_s_ (*d*_ij_ ≤ *h*_s_), and set to 0 otherwise. Here, *d*_ij_ is Euclidean distance between locations *i* and *j*, and *h*_s_ represents the AIC_c_-optimized bandwidth (18). The local regression model was calculated as Equation 6:


$$Y(u_{i},v_{i})=\beta_{0}(u_{i},v_{i})+\sum \mathrm{\beta k}(u_{i},v_{i})X_{k}+\varepsilon (u_{i},v_{i})$$ (6)

GWR quantifies spatially varying relationships through localized parameter estimation at each geographic coordinate.

#### 2.5.4 Geographically and temporally weighted regression (GTWR) model

The GTWR model was employed to analyze spatiotemporal variations in gonorrhea incidence (*Y*) using location (*u*ᵢ,*v*ᵢ) and time (*t*ᵢ)-varying coefficients. The model may be formulated as Equation 7:


$$Y(u_{i},v_{i},t_{i})=\beta_{0}(u_{i},v_{i},t_{i})+\sum \beta_{k}(u_{i},v_{i},t_{i})X_{k}+\varepsilon (u_{i},v_{i},t_{i})$$ (7)

The spatiotemporal weights were computed using a Gaussian kernel function as follows Equation 8:


$$w(d_{\mathrm{st}})=\exp(-d_{\mathrm{st}}^{2}/h_{\mathrm{st}}^{2})$$ (8)

The spatiotemporal distance is computed as $(d_{\mathrm{st}})=\sqrt{\lambda d_{s}^{2}+\mu d_{t}^{2}}$ , combining scaled spatial (*d*_s_) and temporal (*d*_t_) components. The scaling factors *λ* and *μ* were determined by standardizing the spatial and temporal distances to balance their respective influences in the composite spatiotemporal distance metric. The bandwidth *h*_st_ was optimized via AIC_c_ to ensure proper weighting of proximal observations while maintaining model generalizability. This approach captures the dynamic spatial–temporal relationships between gonorrhea incidence and its determinants (27, 28).

#### 2.5.5 Model comparison and evaluation

The study evaluated model performance using multiple metrics including the coefficient of determination (*R*^2^) to measure explained variance, the adjusted *R*^2^ accounting for model complexity, and the corrected Akaike Information Criterion (AIC_c_) assessing model fit while penalizing overfitting, providing comprehensive assessment of both explanatory power and model parsimony across different modeling approaches.

### 2.6 Software implementation

The study employed an integrated analytical approach using ArcGIS 10.2 for spatial autocorrelation analysis and visualization, along with its GTWR plugin for regression modeling (MLR, TWR, GWR, and GTWR). To detect and evaluate statistically significant spatiotemporal clusters of gonorrhea incidence, the spatiotemporal scan statistic was performed using SaTScan software. Authoritative geographic data from China’s National Geographic Information Public Service Platform (map approval: GS(2024)0650) ensured data quality. All analyses used *α* = 0.05 two-tailed tests, combining GIS and spatial statistics to examine spatiotemporal patterns of gonorrhea incidence.

## 3 Results

### 3.1 Spatiotemporal distribution characteristics of gonorrhea incidence

**Figure 1:**
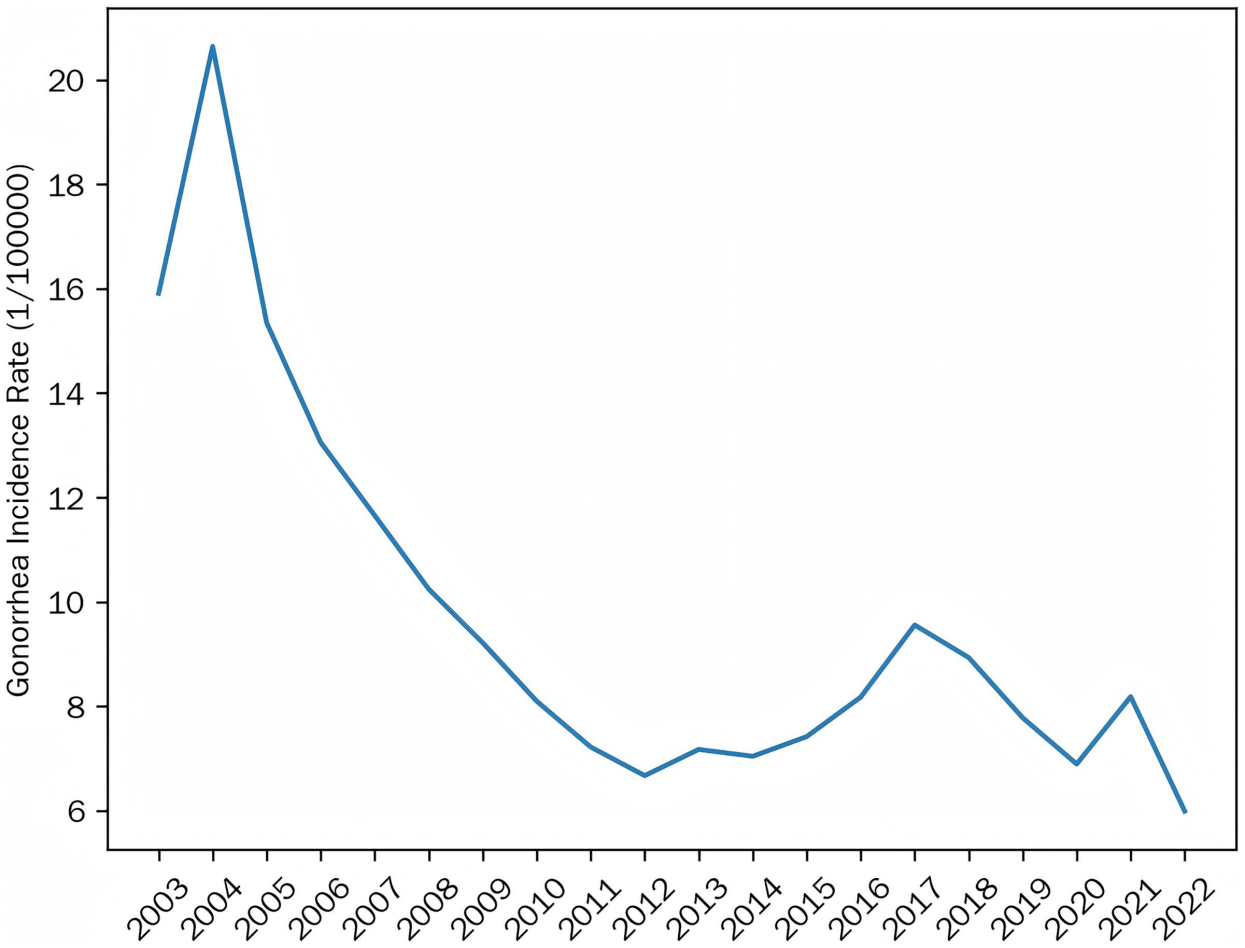
Trends in the average incidence of gonorrhea in China, 2003–2022.

The analysis of average gonorrhea incidence across China’s 31 provinces (2003–2022) revealed clear spatiotemporal patterns. As illustrated in Figure 1, incidence rates showed a consistent decline from 20.64 cases per 100,000 population in 2004 to 5.99 in 2022, though the rate of decrease slowed significantly after 2013.

**Figure 2:**
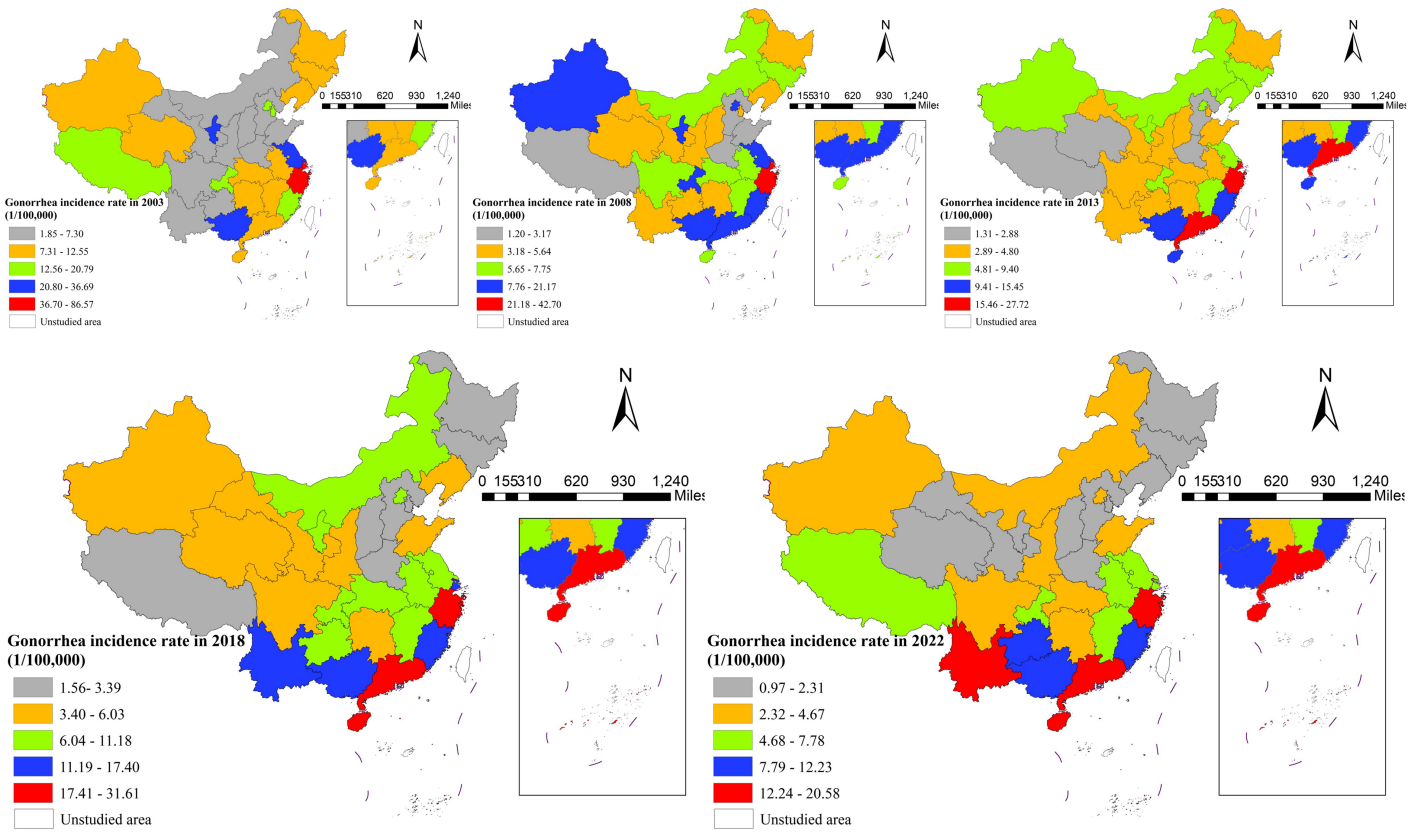
Spatial distribution of gonorrhea incidence in China, 2003, 2008, 2013, 2018, and 2022.

Geographically, the disease distribution underwent notable shifts. High-incidence areas (>36.70/100,000) were concentrated in southeastern coastal provinces in 2003, but contracted substantially by 2008, with emerging hotspots in western regions. By 2022, the spatial pattern had evolved into low-intensity clusters primarily in southern China (Figure 2).

The overall trend showed “decline with localized persistence,” while eastern provinces achieved significant reductions, the southeastern region remained a persistent high-risk area throughout the study period. Conversely, several western provinces (e.g., Yunnan and Guangxi) experienced paradoxical increases in incidence.

### 3.2 Spatial clustering analysis of gonorrhea incidence

The Global Moran’s I analysis revealed significant positive spatial autocorrelation in gonorrhea incidence across China from 2003 to 2022 (all years *p* < 0.05, Table 2). The index values ranged from 0.38 to 0.44, indicating persistent geographic clustering of cases throughout the study period. The spatial autocorrelation peaked at 0.439 in 2009, while the lowest value was 0.380 in 2019. All values were statistically significant and remained consistently above 0.38, demonstrating a stable pattern of spatial concentration. These results suggest that gonorrhea incidence was consistently concentrated in specific geographic regions, with temporal variations that may be influenced by a combination of long-term public health measures and sociodemographic factors.

**Table 2:** Moran’s *I* values for gonorrhea incidence rates (2003–2022).

Year	Moran’s *I*	*p*
2003	0.400498	0.000011
2004	0.406126	0.000002
2005	0.432713	0.000006
2006	0.393976	0.000056
2007	0.399700	0.000055
2008	0.423906	0.000020
2009	0.439422	0.000011
2010	0.429833	0.000013
2011	0.424800	0.000020
2012	0.411261	0.000047
2013	0.437421	0.000017
2014	0.400794	0.000060
2015	0.420586	0.000021
2016	0.436625	0.000017
2017	0.403849	0.000064
2018	0.384218	0.000196
2019	0.380595	0.000262
2020	0.405166	0.000120
2021	0.413482	0.000095
2022	0.391238	0.000204

### 3.3 Spatiotemporal scan analysis results

**Figure 3:**
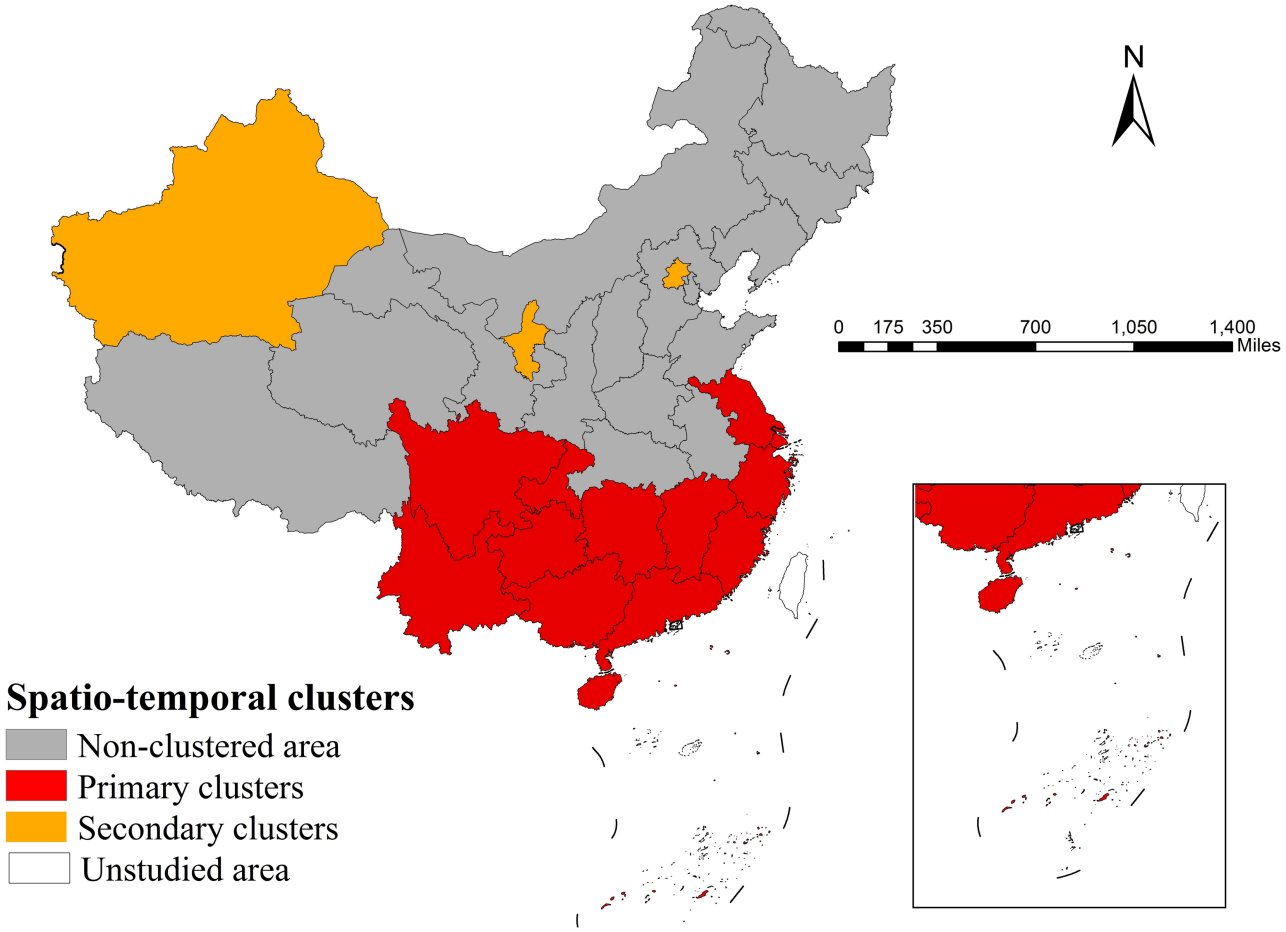
Spatiotemporal cluster regions of gonorrhea incidence in China.

The spatiotemporal scan analysis identified persistent primary clusters in the Yangtze River Delta (Shanghai, Zhejiang, Jiangsu; RR = 4.43) lasting 8 years and southern China (Guangdong, Guangxi plus 9 other provinces; RR = 2.29) lasting 10 years, along with more variable secondary clusters including Beijing (RR = 2.57) for 5 years, northwest regions (Ningxia, Xinjiang; RR = 3.04) and southwest areas (Chongqing, Guizhou; RR = 1.15) each lasting 1 year (Table 3). The results demonstrated stable high-risk zones in eastern and southern coastal regions alongside transient clusters in northern and western interior provinces (Figure 3).

**Table 3:** Results of spatial – temporal scan analysis: regional and temporal distribution of aggregation areas.

Aggregation area	Region	RR	LLR	*p*	Time
Type-I aggregation area	Shanghai, Zhejiang, Jiangsu	4.43	291616.98	<0.001	From 2003/1/1 to 2010/12/31
	Fujian, Jiangxi, Zhejiang, Guangdong	2.29	121917.85	<0.001	From 2012/1/1 to 2021/12/31
	Yunnan, Guizhou, Sichuan, Guangxi, Chongqing, Hunan, Hainan, Guangdong	1.46	15886.03	<0.001	From 2004/1/1 to 2008/12/31
Type-II aggregation area	Beijing	2.57	6326.79	<0.001	From 2003/1/1 to 2007/12/31
	Ningxia Hui Autonomous Region	3.04	3865.44	<0.001	From 2003/1/1 to 2007/12/31
	Xinjiang Uygur Autonomous Region	1.62	966.39	<0.001	From 2006/1/1 to 2008/12/31
	Chongqing and Guizhou	1.15	69.23	<0.001	From 2021/1/1 to 2021/12/31

### 3.4 Multicollinearity testing and model comparison

As shown in Tables 4, 5, initial analysis revealed multicollinearity (the VIF value of urbanization rate was 7.648). After removing this variable, all remaining predictors exhibited acceptable VIF values below 5. Among the four models evaluated, GTWR demonstrated the strongest performance with an adjusted *R*^2^ of 0.8697 and AIC_c_ of 3785.42, followed by GWR (adjusted *R*^2^ = 0.6806, AIC_c_ = 4172.76), TWR (adjusted *R*^2^ = 0.6015, AIC_c_ = 4231.79), and MLR (adjusted *R*^2^ = 0.2200, AIC_c_ = 4573.80) (Table 6). Significant spatial autocorrelation confirmed the necessity of spatial modeling approaches. The GTWR model was ultimately selected for final analysis as it most effectively captured both spatial and temporal variations in gonorrhea incidence patterns, significantly outperforming alternative modeling approaches.

**Table 4:** VIF values of each variable.

Factors	VIF
Number of licensed physicians per 1,000 population	3.727
Hospital bed occupancy rate	1.272
GDP per capita	3.789
Average years of schooling	4.858
Population density	1.733
Average household size	2.802
Sex ratio	1.185
Urbanization rate	7.648

**Table 5:** VIF values of each variable after removing urbanization rate.

Factors	VIF
Number of licensed physicians per 1,000 population	3.318
Hospital bed occupancy rate	1.246
GDP per capita	3.426
Average years of schooling	4.023
Population density	1.585
Average household size	2.608
Sex ratio	1.185

**Table 6:** Goodness-of-fit of the four models.

Model fit	GTWR	GWR	TWR	MLR
*R* ^2^	0.871161	0.684219	0.605996	0.229227
Adjust *R*^2^	0.869687	0.680607	0.601490	0.220000
AIC_c_	3785.42	4172.76	4231.79	4573.80

### 3.5 Temporal evolution characteristics of factors

**Figure 4:**
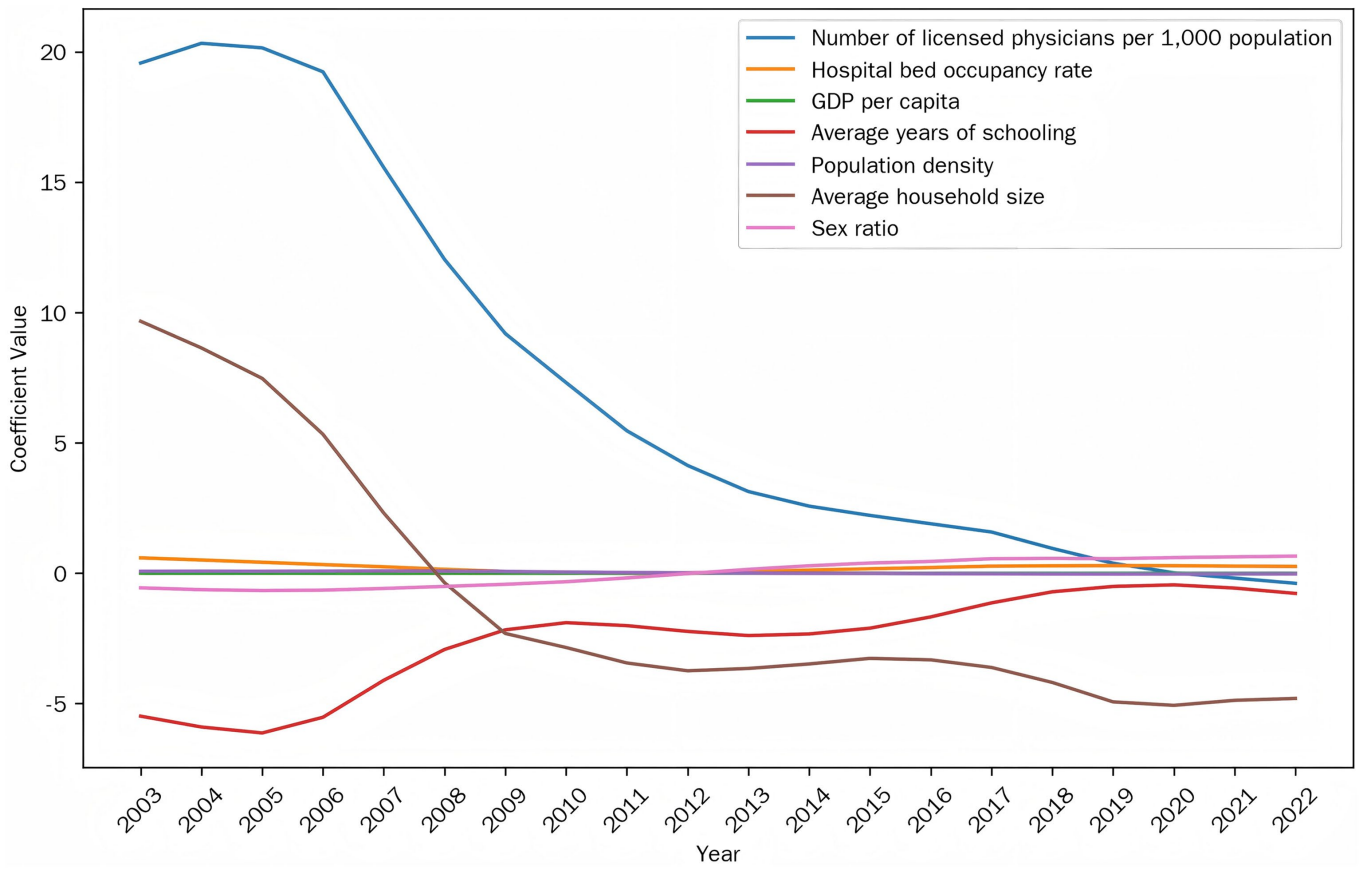
Temporal variations in average regression coefficients of all factors.

Figure 4 shows temporal changes in average regression coefficients for all variables. Medical resources (physicians of 1,000 population, bed occupancy rates) showed weakening positive associations over time, while economic development shifted from protective to risk-enhancing effects, and education maintained stable protective effects. Demographic factors exhibited notable reversals: population density and household size shifted from positive to negative correlations, while gender ratio changed from protective to risk-enhancing. These dynamic patterns reflect China’s socioeconomic transformation and suggest prevention strategies require regular reassessment as underlying risk factor relationships continue evolving.

### 3.6 Spatial heterogeneity of factors

**Figure 5:**
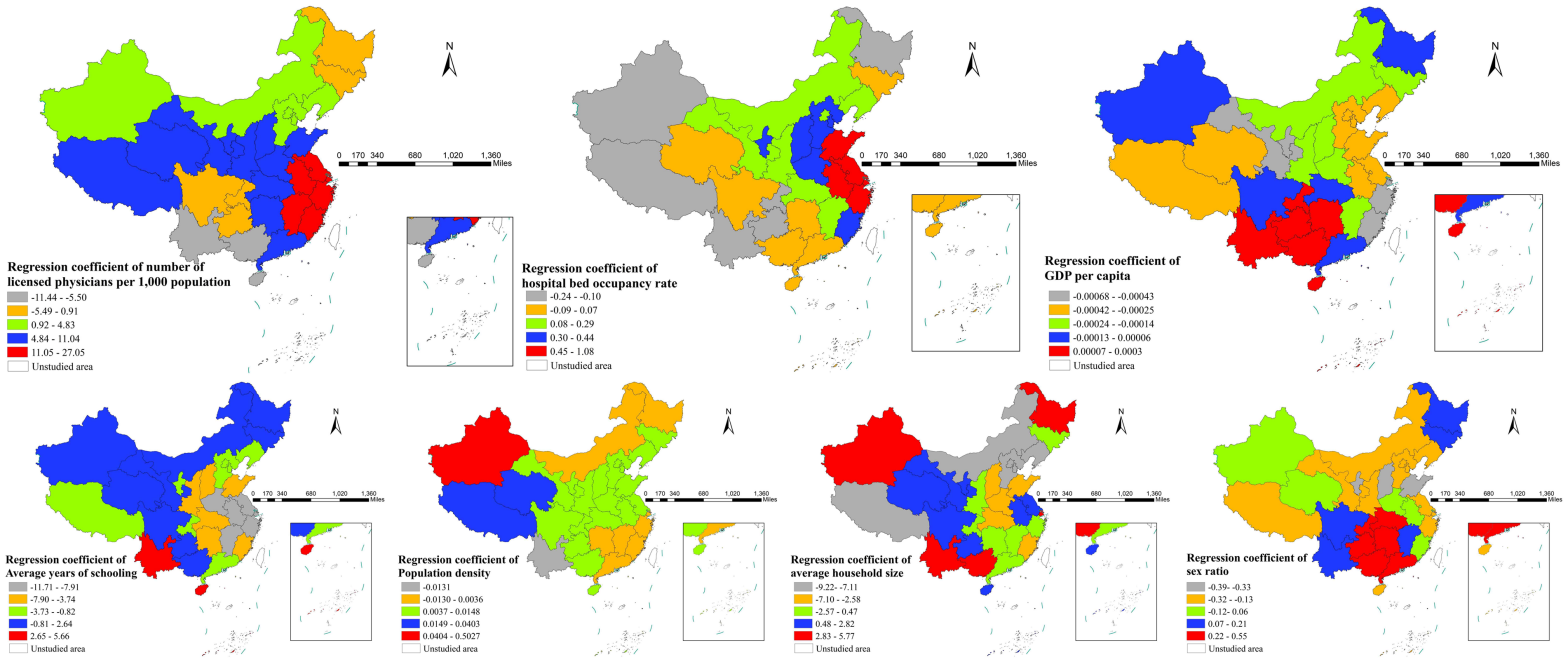
Geographic distribution of average regression coefficients across study factors.

Figure 5 displays the spatial variations in average regression coefficients for all variables. The impact of medical resource availability on incidence exhibited regional variation: positive in eastern coastal provinces and negative in western regions. Economic development and educational attainment showed consistent protective effects in most regions nationwide, with the strongest economic impacts observed in northern China and the most pronounced educational effects concentrated in the Yangtze River basin. Population density showed consistently positive associations with incidence across most regions, with the strongest effects observed in western China. Family structure exhibited regional variations: larger household size increased risk in western regions but decreased risk in northern China, and male-biased gender ratios showed strongest associations with higher incidence in southern provinces. These spatial patterns highlight how regional disparities in development, healthcare access, and demographic structures create distinct epidemiological contexts requiring tailored prevention approaches across different parts of China.

### 3.7 Spatiotemporal dynamics of factors

**Figure 6:**
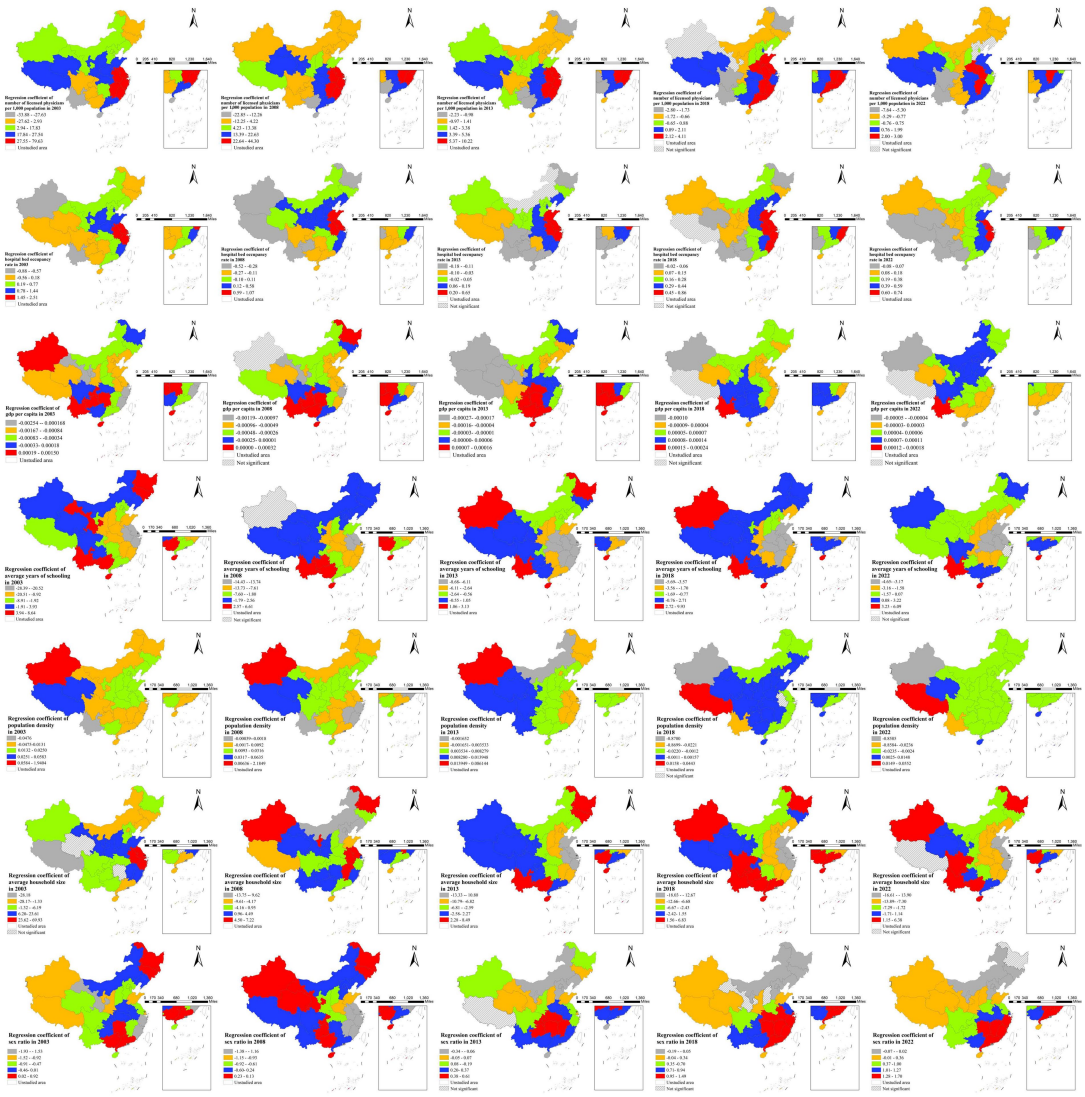
Dynamic spatiotemporal patterns of gonorrhea incidence drivers, 2003, 2008, 2013, 2018, and 2022.

As shown in Figure 6, the integrated spatiotemporal analysis uncovered complex evolving geographical patterns in how various factors influenced gonorrhea transmission from 2003 to 2022. Medical resource indicators maintained persistent east–west gradients in their effects. Economic factors underwent notable regional shifts – with protective effects concentrated in eastern China during 2003–2008, transitioning to western predominance after 2013. Educational attainment consistently showed strongest protective effects in eastern regions throughout the study period. Demographic factors exhibited diverse spatiotemporal trajectories – population density maintained consistently stronger positive associations in western China, while household size effects shifted from eastern to western predominance after 2013. Gender ratio impacts evolved from initially irregular spatial patterns to a clear north–south gradient in later years, with male predominance becoming increasingly associated with higher incidence in southern provinces. These dynamic spatial patterns reflect the complex interplay between China’s uneven regional development, internal migration patterns, and evolving social determinants of health over the study period, emphasizing the need for surveillance systems capable of detecting such epidemiological transitions.

## 4 Discussion

Despite the overall national decline, the paradoxical rise in gonorrhea incidence in several western provinces (e.g., Yunnan and Guangxi) underscores the complexity of regional epidemic control. This disparity likely stems from multifaceted challenges, including socioeconomic factors and limitations in the current healthcare system. To address this gap, a reinforced and tailored strategy is imperative. This may involve optimizing resource allocation, enhancing the accessibility of STI services, and integrating health education with local community programs in western China.

Our study revealed persistent gonorrhea hotspots in China (2003–2022), with elevated risk in the Yangtze River Delta and southern China, aligning with prior reports on eastern provinces like Zhejiang and Guangdong (3, 29). The stronger spatial autocorrelation [Moran’s *I*: 0.38–0.44 vs. 0.197–0.295 in regional studies (3)] underscores nationwide disparities. The southeastern region’s high incidence reflects known drivers such as economic activity and migrant populations, while rising rates in western provinces may stem from healthcare access barriers (2). These patterns call for tailored interventions: targeted migrant health programs in coastal hubs and improved resource allocation in underserved regions.

The GTWR model demonstrated superior performance, significantly outperforming GWR, TWR and MLR models. This aligns with multiple studies highlighting GTWR’s advantages in capturing spatiotemporal heterogeneity (28, 30, 31). The GTWR model successfully integrated spatial and temporal dimensions, with its excellent performance likely stemming from synergistic capture of “spatial heterogeneity and temporal dynamics” (32), providing methodological reference for future spatial epidemiological studies of infectious diseases.

The temporal variation characteristics of gonorrhea incidence determinants were revealed in this study. The diminishing influence of healthcare resource indicators over time may reflect China’s healthcare system transition from quantitative expansion to quality improvement (33). Meanwhile, the stable and consistently protective effect of education stands in contrast to the risk-enhancing role of economic development, reaffirming the need to carefully evaluate the distinct impacts of socioeconomic factors on disease control (34). The differential evolution patterns of demographic factors are particularly noteworthy: shifts in population density and household size effects may relate to accelerated urbanization and nuclear family trends (35), while the reversal of gender ratio influence might reflect changing sexual behavior patterns amid population mobility (36). These dynamic changes suggest gonorrhea prevention strategies require periodic adjustment according to socioeconomic characteristics at different development stages, particularly during rapid demographic transitions when close monitoring of risk factor changes becomes crucial (37).

Significant geographical heterogeneity was revealed in gonorrhea transmission across China. Healthcare accessibility showed strong positive associations with incidence in eastern coastal provinces (2, 3), which can be attributed to better case detection and reporting systems in these well-resourced regions, whereas under-reporting in western China due to limited medical infrastructure may have obscured the true burden. This pattern is further compounded by high population mobility and commercial sex activity in eastern areas. Economic development and education showed consistent protective effects across most of the country, with strongest economic impacts in northern China (2) and most pronounced educational benefits along the Yangtze River, consistent with the region’s concentrated higher education resources. Population density exhibited particularly strong positive correlations in western regions (2), reflecting amplified transmission in densely populated areas. Family structure displayed distinct regional patterns: traditional extended households increased risk in the west, while nuclear families showed protection in the north (2). Notably, male-biased sex ratios in southern provinces correlated strongly with higher incidence (2, 38), possibly due to gender imbalance-driven commercial sex. These findings support regionally tailored strategies: enhancing STI services in eastern coastal areas, behavioral interventions in western population centers, and targeted surveillance in southern provinces with skewed sex ratios (2, 3).

The analysis reveals significant spatial heterogeneity and temporal variations in risk factor effects. Healthcare resources demonstrated persistent east–west gradient impacts, reflecting structural inequities in health infrastructure that may exacerbate regional transmission disparities, as documented in studies of China’s tiered medical system (2). Economic influences underwent marked spatial transitions, showing protective effects initially concentrated in eastern China (2003–2008) before shifting to western dominance post-2013, a pattern that aligns with Chinese Western Development Strategy and subsequent rural-to-urban migration trends, which altered sexual networks and economic vulnerability profiles (2, 39). Education maintained consistently strongest protective effects in eastern regions, indicating its durable positive influence on health behaviors (38). Population factors exhibited diverse spatiotemporal trajectories: population density showed sustained positive associations in western areas, likely amplifying transmission risks (2, 40); household size effects transitioned east-to-west after 2013, possibly associated with urbanization and the fragmentation of traditional household structures in inland provinces (39); while gender ratio impacts evolved into a north–south gradient, with male predominance increasingly associated with elevated incidence in southern provinces, a trend supported by demographic studies highlighting the concentration of unmarried male migrants in industrial zones of southern China, which may foster commercial sex networks (2, 29). These dynamic patterns underscore the complex interplay between China’s regional disparities, population mobility, and evolving social determinants in shaping transmission dynamics.

To address the spatial heterogeneity and spatiotemporal dynamics observed, region-specific prevention strategies are recommended. In eastern coastal provinces, optimizing the allocation and utilization of medical resources should be prioritized, while western regions require enhanced healthcare infrastructure. Northern China may focus on leveraging economic growth for health investment, whereas the Yangtze River basin should strengthen health education. Southern provinces need targeted interventions addressing male-biased gender ratios, and western regions should manage risks associated with population density and larger household sizes. Additionally, dynamic surveillance systems must be established to monitor evolving risk patterns and adjust strategies accordingly.

This research presents an innovative methodological approach that combines spatiotemporal scan statistics with GTWR modeling to elucidate the complex dynamics of gonorrhea transmission in China. The study makes three key contributions: (1) identification of persistent high-risk clusters in the Yangtze River Delta and southern regions, along with emerging hotspots in western areas; (2) pioneering quantification of spatiotemporal variations in influencing factors; and (3) development of a comprehensive 20-year provincial database (2003–2022) for robust epidemiological analysis. These advances establish a valuable framework for infectious disease surveillance and inform targeted prevention strategies.

Some limitations merit consideration. The provincial-level analysis may not fully capture local transmission patterns due to data resolution constraints. Furthermore, the exclusion of data on population mobility, behavioral data (e.g., high-risk sexual behavior and condom use), and health system factors such as regional testing rates or reporting completeness limits the completeness of our findings and may influence both the location and magnitude of the detected clusters. Therefore, the results should be interpreted with caution in this context. Future research incorporating more granular data and additional variables would further enhance understanding of disease transmission dynamics. Finally, while the superior performance of the GTWR model is supported by both high adjusted R2 and low AICc, its validation was based on in-sample fit. Thus, employing cross-validation in future studies remains valuable to further confirm its predictive power.

## 5 Conclusion

This study elucidates the spatiotemporal patterns and underlying drivers of gonorrhea incidence in China (2003–2022) through integrated spatiotemporal scan statistics and GTWR modeling. The results reveal a “general decline with localized persistence” trend, characterized by significant reductions in eastern regions contrasting with sustained high incidence in the southeastern region and emerging hotspots in western provinces. Our analyses demonstrate temporally weakening healthcare resource effects, a shift in economic development from protective to risk-enhancing effects, and stable protective effects of education, highlighting the complex and evolving interplay between regional development disparities and disease transmission dynamics. These findings underscore the necessity for geographically tailored prevention strategies, prioritizing persistent high-risk clusters while monitoring emerging areas. The study provides a useful analytical framework for infectious disease surveillance and offers data-driven insights to inform targeted prevention strategies.

## Data Availability

The original contributions presented in the study are included in the article/Supplementary material, further inquiries can be directed to the corresponding author.
